# Locally Advanced Prostate Squamous Cell Carcinoma Diagnosed Using PET‐CT and Treated With Robot‐Assisted Radical Prostatectomy

**DOI:** 10.1002/iju5.70088

**Published:** 2025-08-21

**Authors:** Masayuki Waki, Akira Fujisaki, Shunya Usami, Kei Muraoka, Yasuhiro Hakamata, Yuka Kanda, Kota Sugiura, Masashi Yoshida, Yoshiro Otsuki, Tatsuaki Yoneda

**Affiliations:** ^1^ Department of Urology Seirei Hamamatsu General Hospital Hamamatsu Japan; ^2^ Department of Pathology Seirei Hamamatsu General Hospital Hamamatsu Japan

**Keywords:** genetic panel testing, multimodal treatment, PET‐CT, RARP, squamous cell carcinoma of the prostate

## Abstract

**Introduction:**

Primary squamous cell carcinoma of the prostate accounts for < 1% of prostate cancers.

**Case Presentation:**

A 70‐year‐old man with no urinary symptoms and a normal prostate‐specific antigen of 0.96 ng/mL was referred for abnormal prostate uptake on positron emission tomography‐computed tomography. He was diagnosed with locally advanced squamous cell carcinoma of the prostate with lymph node metastasis. Robot‐assisted radical prostatectomy and adjuvant chemoradiotherapy were administered. The patient remained in complete remission at 18 months postoperatively.

**Conclusion:**

Squamous cell carcinoma of the prostate should be suspected based on positron emission tomography‐computed tomography prostate uptake. Robot‐assisted radical prostatectomy may be feasible for locally advanced squamous cell carcinoma of the prostate; genetic profiling should be considered to identify targeted therapies.

**Summary**

- This novel case highlights the critical role of positron emission tomography‐computed tomography in identifying rare and aggressive malignancies such as primary squamous cell carcinoma of the prostate (SCCP), even in asymptomatic patients with normal PSA levels.
- The locally advanced SCCP with lymph node metastasis was successfully treated with robot‐assisted radical prostatectomy followed by multimodal therapy, achieving complete remission at 18 months.

**Abbreviations**
5‐FU: fluorouracil
CDDP: cisplatin
CR: complete remission
CT: computed tomography
MRI: magnetic resonance imaging
PET‐CT: positron emission tomography‐computed tomography
PSA: prostate‐specific antigen
RARP: robot‐assisted radical prostatectomy
SCC: squamous cell carcinoma
SCCP: squamous cell carcinoma of the prostate
TMB: tumor mutational burden

## 1 Introduction

Primary squamous cell carcinoma (SCC) of the prostate (SCCP) is a rare and aggressive malignancy, accounting for < 1% of prostate cancers [1, 2]. Patients present with nonspecific symptoms such as urinary obstruction or retention, with normal serum prostate‐specific antigen (PSA) levels [1, 3]. SCCP is frequently diagnosed at advanced stages [4].

Multimodal treatment may improve outcomes in SCCP [2]. Combining local therapy with chemotherapy can extend median survival for localized disease by 12 to 29 months [1]. However, prognosis remains poor, with a median overall survival of 14 months [1].

**FIGURE 1:**
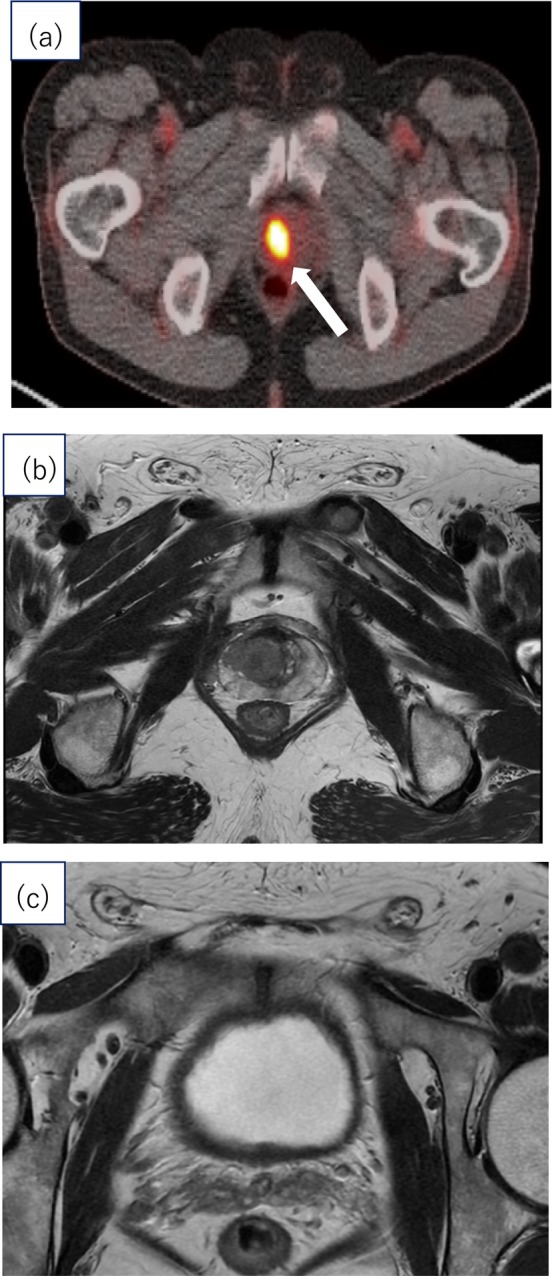
Findings in the Prostate Region: Positron emission tomography‐computed tomography and Magnetic resonance imaging. (A) Abnormal uptake in the right lobe of the prostate on PET‐CT (white arrow). PET‐CT showed no significant FDG uptake in the lymph node region. (b) MRI showed a 15‐mm tumor in the right lobe of the transition zone of the prostate invading beyond the capsule Low signal intensity on T2‐weighted images. (c) MRI revealed no findings suggestive of seminal vesicle invasion, nor were there any findings indicative of pelvic lymph node metastasis on T2‐weighted images.

We report a rare case of SCCP with lymph node metastasis diagnosed by positron emission tomography‐computed tomography (PET‐CT) (Figure 1a). The patient was treated with robot‐assisted radical prostatectomy (RARP), radiotherapy, and chemotherapy, achieving complete remission (CR) for 18 months.

## 2 Case Presentation

**FIGURE 2:**
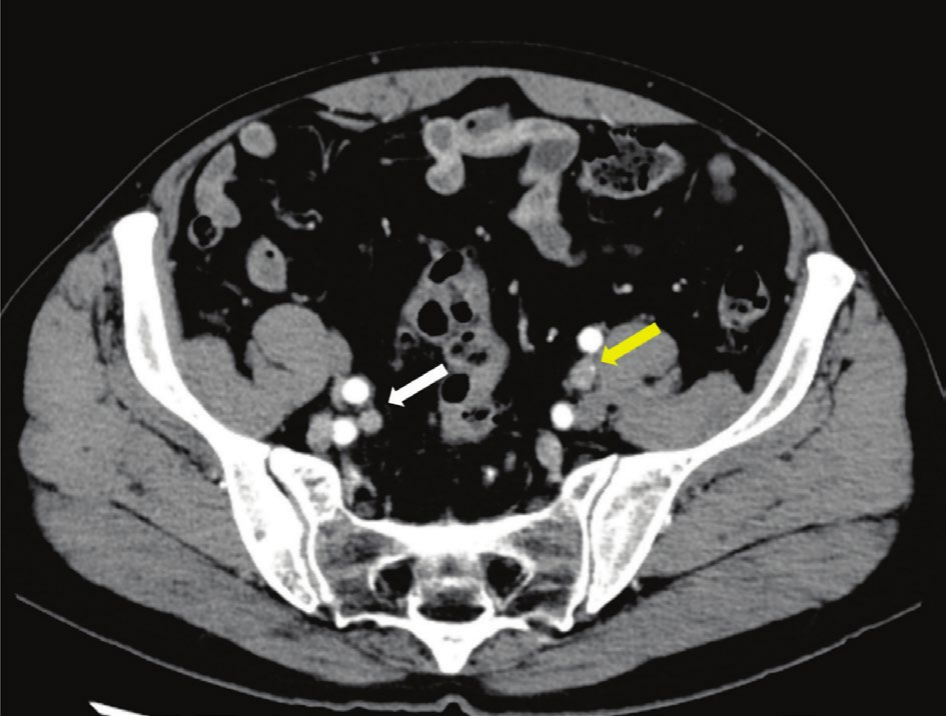
Contrast‐enhanced CT. Contrast‐enhanced CT revealed bilateral enlargement of the external iliac lymph nodes (white arrow, right external iliac lymph node; yellow arrow, left external iliac lymph node).

The patient was a 70‐year‐old man with no history of cancer or prior surgeries. Incidental prostate uptake was observed on PET‐CT performed during a health checkup for non‐urological screening purposes. When he presented to our hospital, the PSA level was within the normal range (0.96 ng/mL). Digital rectal examination detected a nodule in the right lobe. MRI revealed a 15‐mm lesion in the right transition zone (Figure 1b,c). SCC was diagnosed via biopsy; pathological findings showed no glandular formation. Immunohistochemistry was negative for PSA and GATA3 and positive for p40. Serum SCC antigen level was elevated (3.8 ng/mL). Contrast‐enhanced CT revealed bilateral external iliac lymph node metastasis (Figure 2), with no evidence of a primary SCC lesion outside the prostate. Bone scintigraphy revealed no metastasis. Preoperative urine cytology was negative. The patient was diagnosed with SCCP (cT3aN1M0).

**FIGURE 3:**
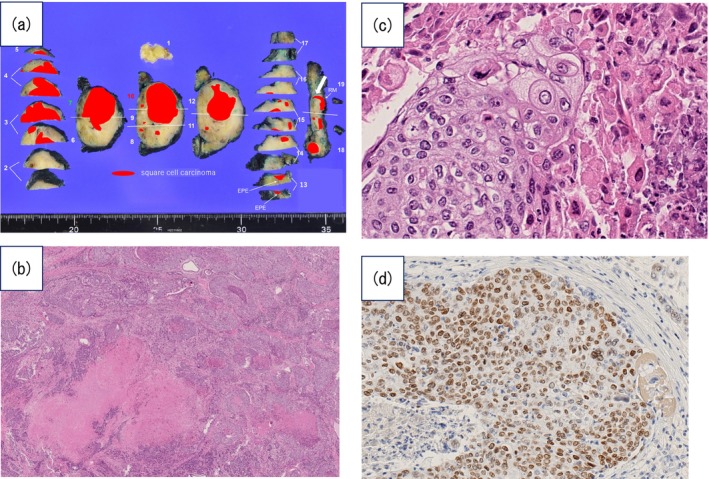
Pathological findings of the resected prostate. (a) The resected specimen included the prostate and seminal vesicles. Positive margin noted at the right seminal vesicle (white arrow). (b) Low magnification shows a diffuse proliferation of tumor cells with marked necrosis. No glandular structures were observed in the tumor area. No intraepithelial lesion (urothelial or squamous dysplasia/metaplasia) was observed in the prostate. (HE, ×2). (c) High magnification reveals that the tumor grows in nests, with tumor cells exhibiting intercellular bridges and keratinization. (HE, ×40). (d) Immunohistochemical expression of p40 suggests squamous cell carcinoma. (×20)

RARP with pelvic lymph node dissection (external iliac, internal iliac, and obturator lymph nodes) was performed. Pathological examination revealed SCC with lymphovascular invasion, seminal vesicle invasion, and a positive surgical margin at the right seminal vesicle. Immunohistochemical and histological analyses showed no evidence of urothelial carcinoma or prostatic adenocarcinoma, and no carcinoma in situ was identified (Figure 3). No intraepithelial lesion (urothelial carcinoma in situ, urothelial dysplasia, squamous dysplasia, or squamous metaplasia) was identified in the prostatic urethral mucosa. No urothelial dysplasia, squamous dysplasia, or squamous metaplasia was identified. Of 30 dissected lymph nodes, six exhibited metastasis. Postoperatively, the SCC antigen normalized to 0.9 ng/mL. Genomic profiling using FoundationOne CDx revealeda tumor mutational burden (TMB) of 11 mutations per megabase and pathogenic alterations in *PTEN* (p.Q171*) and *TP53* (p.H179Y).

On postoperative day 21, radiation to the pelvis and prostate bed (45 Gy/25 fractions) was initiated concurrently with adjuvant chemotherapy using fluorouracil (5‐FU) and cisplatin (CDDP). The patient developed strangulated ileus on radiotherapy day 15, requiring surgery and radiation discontinuation. Chemotherapy was resumed but discontinued after the third cycle because a 5‐FU‐induced erythema multiforme‐like drug eruption occurred. The patient has been under observation since then. At 18 months postoperatively, CT showed no recurrence and normal SCC antigen level, indicating a CR.

## 3 Discussion

The Surveillance, Epidemiology, and End Results database identified 66 cases between 2000 and 2018, with a median age of 67 years (range: 35–85 years) [1]. Stages at diagnosis are localized (24.2%), locally advanced (22.7%), distant metastasis (28.8%), and unknown stage (24.2%) [4]. Common metastatic sites include bone (33%), lymph nodes (30%), lungs (20%), liver (7%), and brain (7%) [4]. The overall 5‐year survival rate for SCCP is 24% [1].

To our knowledge, this is the first report of incidental diagnosis of SCCP using PET‐CT in an asymptomatic patient. PET‐CT is valuable in diagnosing and managing SCCP [5, 6, 7, 8, 9, 10], as it exhibits high FDG uptake, enabling clear visualization of primary and metastatic lesions. Post‐treatment PET‐CT assesses therapeutic response [5, 10]. SCCP should be considered for patients with normal PSA levels if PET‐CT shows increased prostate uptake.

The prostate lacks native squamous epithelium; squamous metaplasia may arise secondarily in the settings of chronic inflammation or prior therapy [11]. Several mechanisms for SCCP exist, including therapy‐related trans differentiation of adenocarcinoma and derivation from basal/pluripotent progenitor cells [11]. Here, diffuse keratinizing squamous morphology without glandular structures, absence of intraepithelial lesions in the prostate/prostatic urethra, and an immunohistochemical profile favored a primary prostatic origin, while acknowledging that immunohistochemistry alone is not definitive. FDG PET‐CT demonstrated intense uptake confined to the prostate without identifying an alternative primary site and was useful for staging and response assessment [5, 7, 10]. The lack of pathological assessment of the bladder mucosa is a limitation; cystoscopic surveillance with biopsy will be performed if clinically indicated.

The treatment for SCCP remains unclear. Multimodal treatments improve patient survival. For locally advanced SCCP, combining local treatment with chemotherapy provides better survival than chemotherapy alone (median survival: 37 vs. 11 months) [12]. However, whether surgery or radiation is more beneficial remains unclear as no significant difference in survival was observed (*p* = 1.0) [1].

Table 1 presents reported cases of SCCP. Radiotherapy with chemotherapy is the most used local treatment. Prostatectomy has never been reported. No previous reports of RARP being used to treat locally advanced SCCP exist. We chose RARP, a minimally invasive surgical approach, because no preoperative finding suggested bladder invasion, and cystoprostatectomy is excessively invasive. Also, tumor volume reduction could improve the efficacy of adjuvant chemotherapy. Local control was achieved with adjuvant radiotherapy. SCC is radiosensitive, though evidence for SCCP is limited. Here, the tumor responded well without cystectomy. We selected surgery first over radiotherapy to enhance adjuvant treatment and minimize tumor burden. As no standard chemotherapy exists for SCCP, maximal tumor reduction before adjuvant therapy was essential.

**TABLE 1:** Clinical characteristics and treatment outcomes of reported cases of locally advanced SCCP.

Authors	age	PSA (ng/mL)	cTNM	Chief complaint	Diagnosis	Local treatment	Systematic treatment	Follow (month)	Prognosis
Imamura et al. (2000) [13]	54	1.7	T4N0M0	Dysuria	Biopsy	Cystoprostatectomy	Adjuvant: Methotrexate + Peplomycin + CDDP +5‐FU	60+	Alive, CR
Okada et al. (2000) [14]	65	1.1	T3N1M0	Acute urinary retention	Biopsy	Radiation 60Gy (Whole pelvis 50Gy + Prostate 10Gy)	Peplomycin + CDDP	18+	Alive, CR
Sudhakar et al. (2004) [15]	29	4	T4N0M0	Dysuria, constipation, weight loss	Biopsy	Radiation (NS)	No	NS	NS
Kanthan et al. (2004) [16]	77	NS	Stage C	Obstruction	Biopsy	TURP	Chemo (NS)	5	Dead
	42	NS	Stage C	Urinary retention	TURP	No	No	2	Dead
	82	NS	Stage C	Urinary infection, obstruction	Biopsy	No	No	1	Dad
Munoz et al. (2007) [2]	76	NS (normal)	T3aN0M0	Acute urinary retention	Biopsy	Radiation 72Gy (Whole pelvis 46Gy + Prostate bed 20Gy + Prostate gland 6Gy)	5‐FU + CDDP	60	Relapsed, Dead
Raheem et al. (2009) [17]	74	19	T4N1M0	Hematuria, urinary retention	TURP	Radiation 18Gy (discontinued; fistula) + ileal conduit and sigmoid colostomy	No	12+	Alive
Kara et al. (2014) [7]	77	4.3	TxN1M0	Decreased urinary output	TURP	Radiation 76.4Gy (Whole pelvis 50.4Gy + Prostate 16Gy + Lymph node 10Gy)	No	NS	NS
Biswas et al. (2015) [5]	58	1.2	T4N1M0	Anorectal discomfort, hematochezia	Biopsy	Radiation 54Gy (Whole pelvis 45Gy + Prostate 9Gy)	MMC + 5‐FU	27+	Alive, CR
Onoda et al. (2017) [12]	55	0.31	T3bN1M0	Dysuria, gross hematuria	Biopsy	Radiation 64Gy (Whole pelvis + Prostate)	Docetaxel +5‐FU + CDDP	24+	Alive, CR
Atagi et al. (2021) [6]	74	1.62	T4N1M0	Frequent urination	Biopsy	Radiation 78Gy (Whole pelvis + Prostate)	Docetaxel +5‐FU + CDDP	6+	Alive, CR
Tahbaz et al. (2022) [18]	57	NS	T4N1M0	Hematuria, urinary frequency	Open prostatectomy	Cystoprostaurethrectomy + Adjuvant radiation (NS)	Adjuvant: NS	NS	Alive
Present case	70	0.96	T3aN1M0	PET‐CT (asymptomatic)	Biopsy	RARP + Adjuvant radiation 27Gy (discontinued; ileus)	Adjuvant: 5‐FU + CDDP	18+	Alive, CR

Abbreviations: CDDP, cisplatin; CR, complete response; 5‐FU, fluorouracil; MMC, mitomycin C, NS, not stated.

Cisplatin‐based regimens are commonly administered for SCCP. Long‐term survival has been reported with CDDP and 5‐FU [2, 19]. Favorable outcomes have also been reported with docetaxel, CDDP, and 5‐FU [6, 10]. The efficacy of neoadjuvant or adjuvant chemotherapy remains unclear [1]. Based on previous reports, we selected CDDP and 5‐FU regimens. Given the uncertain response rates and limited second‐line options, we opted for adjuvant rather than neoadjuvant chemotherapy.

Next‐generation sequencing (NGS) identifies therapeutic options for SCCP during recurrence. NGS is covered by insurance for solid tumors lacking standard treatments or for patients nearing the end of standard therapy. A previous study identified SMARCA4 (p.D1235E) [20]. Genetic testing revealed a high tumor mutational burden (TMB; 11 mutations/Mb), PTEN truncation (p.Q171*), and a *TP53* missense mutation (p.H179Y). If recurrence occurs, pembrolizumab is used owing to the high TMB. Given the poor prognosis and lack of effective second‐line chemotherapy, NGS is recommended for SCCP.

## 4 Conclusion

This case highlights the potential utility of PET‐CT in detecting SCCP and supports the feasibility of RARP as a treatment option. Owing to its aggressive nature, an effective multimodal treatment strategy is necessary, making RARP a feasible option for locally advanced SCCP. Furthermore, genetic profiling may provide valuable insights into potential targeted therapies.

## Consent

Written informed consent was obtained from the patient for the publication of this report.

## Conflicts of Interest

The authors declare no conflicts of interest.
